# γ-Glutamylcysteine Exerts Neuroprotection Effects against Cerebral Ischemia/Reperfusion Injury through Inhibiting Lipid Peroxidation and Ferroptosis

**DOI:** 10.3390/antiox11091653

**Published:** 2022-08-25

**Authors:** Ruyi Zhang, Jianzhen Lei, Luyao Chen, Yanan Wang, Guocui Yang, Zhimin Yin, Lan Luo

**Affiliations:** 1 State Key Laboratory of Pharmaceutical Biotechnology, School of Life Sciences, Nanjing University, Nanjing 210023, China; 2 Jiangsu Province Key Laboratory for Molecular and Medical Biotechnology, College of Life Science, Nanjing Normal University, Nanjing 210046, China

**Keywords:** γ-glutamylcysteine, cerebral ischemia/reperfusion injury, glutathione, glutathione synthetase, ferroptosis, lipid peroxidation

## Abstract

Ferroptosis is a non-apoptotic form of cell death driven by iron-dependent lipid peroxidation. Recent evidence indicates that inhibiting ferroptosis could alleviate cerebral ischemia/reperfusion (CIR) injury. γ-glutamylcysteine (γ-GC), an intermediate of glutathione (GSH) synthesis, can upregulate GSH in brains. GSH is the co-factor of glutathione peroxidase 4 (GPX4), which is the negative regulator of ferroptosis. In this study, we explored the effect of γ-GC on CIR-induced neuronal ferroptosis and brain injury. We found that γ-GC significantly reduced the volume of cerebral infarction, decreased the loss of neurons and alleviated neurological dysfunction induced by CIR in rats. Further observation showed that γ-GC inhibited the CIR-caused rupture of the neuronal mitochondrial outer membrane and the disappearance of cristae, and decreased Fe^2+^ deposition and lipid peroxidation in rat cerebral cortices. Meanwhile, γ-GC altered the expression of some ferroptosis-related proteins in rat brains. Mechanistically, γ-GC increased the expression of GSH synthetase (GSS) for GSH synthesis via protein kinase C (PKC)ε-mediated activation of nuclear factor erythroid 2-related factor (Nrf2). Our findings suggest that γ-GC not only serves as a raw material but also increases the GSS expression for GSH synthesis against CIR-induced lipid peroxidation and ferroptosis. Our study strongly suggests that γ-GC has potential for treating CIR injury.

## 1. Introduction

Stroke is a major cause of mortality and adult physical disability worldwide [1]. As the elderly population has expanded, stroke-related death and disability has caused a heavy social and economic burden. The Global Burden of Disease Study 2017 indicated that China had the highest number of prevalent cases of stroke in the world [2]. Almost 80% of strokes are ischemic strokes, the incidence and mortality of which have increased over the past three decades in China [3]. Ischemic stroke is caused by arterial occlusion, leading to a temporary lack of glucose and oxygen supply in the affected brain area [4]. Since the brain is a highly energy-consuming organ, an uninterrupted supply of oxygen and glucose is essential [5]. Thus, ischemia and hypoxia will result in a tissue ischemic damage [6]. Timely restoration of blood flow to ischemic brain areas, such as through thrombolytic treatment, is the primary therapeutic strategy recommended by the current clinical guidelines [7]. However, reperfusion therapy after a period of severe or complete ischemia often cannot reverse but instead enhances tissue damage in the affected brain area, which is called cerebral ischemia/reperfusion injury [8].

During the cerebral ischemia/reperfusion, oxidative stress induced by reactive oxygen species (ROS) accumulation plays a pivotal role in the pathophysiological changes of brain tissue damage [9]. When the blood supply to a region of the brain is blocked, the nutrients and oxygen in those tissues are decreased significantly, leading to the disorder of energy metabolism. As oxygen becomes enriched during blood reperfusion, pro-oxidant enzymes and oxygen as a substrate in mitochondria contribute to the rapid generation of ROS. Large amounts of ROS lead to the breakdown of antioxidant systems, followed by DNA damage, protein dysfunction and lipid peroxidation [10]. Since there are high metabolic rates of oxygen consumption, high levels of polyunsaturated fatty acids (PUFAs) and low antioxidant enzyme activity, neurons are more susceptible to lipid peroxidation than other cells [11].

Ferroptosis is a non-apoptotic form of cell death, which is characterized by excessive iron-dependent lipid peroxidation [12,13]. Abnormal accumulation of labile Fe^2+^ could directly catalyze the formation of free radicals via Fenton chemistry, which leads to intracellular lipid peroxidation Glutathione peroxidase 4 (GPX4), utilizing GSH as a cofactor; this enzyme is required for the clearance of lipid ROS [14]. GSH depletion could result in GPX4 inactivation and subsequent lipid ROS accumulation and ferroptosis [15,16,17]. Previous research has demonstrated that iron overload aggravates brain damage induced by focal ischemia and early reperfusion [18]. Tuo et al. reported that CIR increased iron accumulation and lipid peroxidation in lesioned hemispheres of mice [19]. We suspect that inhibiting lipid peroxidation and ferroptosis may be an effective strategy to alleviate cerebral ischemia/reperfusion injury.

Clinical studies have shown that low levels of GSH are associated with a higher risk of stroke compared with the reduction of other endogenous antioxidants [20]. GSH synthesis involves two consecutive ATP-dependent reactions in cytoplasm. In the first step, glutamate-cysteine ligase (GCL), also called γ-glutamylcysteine synthetase (GCS), catalyzes the reaction between the γ-carboxyl group of glutamate and L-cysteine to γ-glutamylcysteine (γ-GC). GCL is a rate-limiting enzyme, which is feedback-inhibited by GSH. The second step is catalyzed by GSH synthetase (GSS), which links glycine to γ-GC to form GSH [21]. Exogenous GSH is difficult to transport into cells due to an extreme concentration gradient and the hydrolyzed effect of extracellular γ-glutamyl *transpeptidase* (GGT) [22,23]. *N*-acetyl cysteine (NAC) is used to supply intracellular GSH levels, but NAC is first deacetylated to cysteine and is then catalyzed by both GCL and GSS to synthesize GSH [24]. Since cysteine is easily produced in the liver through conversion from the other sulfur-containing amino acids, supplementing with NAC to increase intracellular GSH is of little value [25]. Therefore, finding more effective agents for increasing intracellular GSH remains a critical challenge. Anderson and Meister first reported that γ-GC increases the cellular GSH levels in the kidney [26]. It was reported that the intracerebroventricular administration of γ-GC dose-dependently increases GSH levels in the substantia nigra and in the rest of the brain stem of rats [27]. A single intravenous injection of γ-GC to mice significantly increases GSH levels in the brain in vivo, suggesting that γ-GC is capable of crossing the blood–brain barrier [28]. More recently, it was reported that γ-GC maintains redox control, both in in vitro and in vivo models, by acting as a glutathione peroxidase-1 (GPX1) cofactor. Nuclear factor erythroid 2-related factor (Nrf2) regulates the expression of genes involved in antioxidant defenses [29]. Protein kinase C (PKC) kinases induce Nrf2 phosphorylation, which is established as the upstream signal of Nrf2 activation [30].

Since cerebral ischemia/reperfusion injury is reported to be related with lipid peroxidation and ferroptosis, here we investigated whether γ-GC could inhibit CIR-induced lipid peroxidation and ferroptosis and analyzed the related mechanism.

## 2. Materials and Methods

### 2.1. Animals and Reagents

Male Sprague-Dawley (SD) rats (260–300 g) were purchased from Xipuer-Beikai Experimental Animal Co., Ltd. (Shanghai, China). The animal study was approved by the Medical Laboratory Animal Research Institute of Medical Sciences China (Permit Number: SYXK (Su) 2019-0056). All treatment of rats in this study was in strict agreement with the guidelines on ARRIVE and recommendations from an NIH-sponsored workshop regarding experimental design and reporting standards [31]. Animals were housed at 20–24 °C, with 40–60% humidity in a 12 h light/12 h dark cycle and with food and water ad libitum.

γ-glutamylcysteine (γ-GC) was obtained from Prof. ZM Yin (Nanjing Normal University, China). NAC (CAS: 616-91-1) was purchased from Sigma Aldrich (St. Louis, MO, USA). ML385 (HY-100523), and ε-V1-2 (HY-P0154) was purchased from MedChem Express (Shanghai, China).

### 2.2. Rat Middle Cerebral Artery Occlusion/Reperfusion (MCAO/R) Model

Transient acute focal cerebral ischemia was induced by occlusion of the middle cerebral artery as previously described [32]. Rats were anesthetized by intraperitoneal injection of 1% pentobarbital sodium, and then the right common carotid artery bifurcation was exposed. A monofilament was inserted into the right internal carotid artery through the external carotid artery stump. After 90 min, the monofilament was withdrawn for reperfusion. Rats were randomly divided into four groups, including Sham, MCAO/R, MCAO/R+γ-GC (688 mg/kg) and MCAO/R+NAC (522 mg/kg) groups. Similar procedures but without occlusion of the middle cerebral artery were performed on Sham rats. Rats were administrated with γ-GC and NAC orally at the onset of reperfusion. Twenty-four hours after reperfusion, neural dysfunction was evaluated using the deficit grading system introduced by Longa et al. [33], and brain sections were stained with 2,3,5-triphenylterazolium chloride (TTC, Sigma Aldrich, St. Louis, MO, USA) to evaluate the infarction size after MCAO/R (see Supplementary Methods).

### 2.3. Cell Culture and Oxygen-Glucose Deprivation and Reoxygenation (OGD/R) Model

Primary cortical neurons were harvested from embryonic day 18.5 SD rats as described previously [34]. Cells were cultured in neurobasal medium supplemented with 2% B27 and 1% Glutamax at 37 °C with 5% CO_2_. Rat pheochromocytoma (PC12, CRL172.1^TM^) cells purchased from ATCC (Manassas, USA) were cultured in DMEM supplemented with 10% FBS, 1% penicillin/streptomycin at 37 °C with 5% CO_2_.

The OGD/R model was established as previously described [35]. In brief, cells were incubated using a specialized hypoxia incubator, which contained an anaerobic gas mixture (95% N_2_ and 5% CO_2_) kept at 37 °C. Primary cortical neurons were maintained in glucose-free DMEM medium without FBS, and then the cells were transferred to a hypoxic incubator to incubate at 37 °C for 1 h before reoxygenation. PC12 cells were maintained in glucose-free DMEM medium without FBS, and then the cells were transferred to a hypoxic incubator to incubate at 37 °C for 4 h before reoxygenation. For reoxygenation, the cells were refreshed with normal culture medium under normoxic conditions (95% O_2_ and 5% CO_2_) for 12 h. Control cells were incubated in serum-free DMEM in a normoxic incubator chamber.

### 2.4. Fluoro-Jade B Staining and Immunofluorescence Microscopy

Rats were anesthetized with 1% pentobarbital sodium and perfused intracardially with 4% paraformaldehyde. The rat brains were post-fixed in 4% paraformaldehyde overnight and embedded in paraffin blocks, which were cut into 10 μm-thick coronal sections at 200 μm intervals. The sections were de-paraffinized with xylene followed by rehydration. Fluoro-Jade B (FJB) slices were incubated with 0.06% potassium permanganate solution for 15 min and 0.001% FJB staining solution for 30 min at room temperature. After mounting with an anti-fluorescence quencher, images were visualized with a Leica DM4000B fluorescence microscope (Leica, Germany).

For immunofluorescence staining, brain sections were boiled for 20 min in sodium citrate buffer (0.01 M, pH 6.0) for antigen retrieval after being dewaxed and rehydrated and were blocked with 3% BSA. The slides were incubated with primary antibodies at 4 °C overnight. Antibody information is included in the Supplementary Materials. After being washed three times with PBS, the slides were incubated with the fluorescence-conjugated secondary antibodies at room temperature in the dark for 1 h. Sections were coverslipped with polar mounting anti-fluorescence quencher. Fluorescent signals were viewed using a Leica DM4000B fluorescence microscope (Leica, Wetzlar, Germany).

### 2.5. Confocal Microscopy

PC12 cells were rinsed with PBS three times and were fixed in 4% paraformaldehyde for 15 min at room temperature. After being permeabilized with 0.1% Triton X-100 for 30 min, cells were blocked with 3% BSA for 40 min and incubated with primary antibody overnight at 4 °C. Cells were rinsed with PBS three times and were incubated with FITC-conjugated secondary antibody at room temperature in the dark for 1 h followed by incubating with DAPI solution for 5 min. After being mounted with anti-fluorescence quencher mounting medium, the fluorescent signals were visualized under a Nikon C1 confocal laser microscope (Nikon, Tokyo, Japan).

### 2.6. Immunoblotting and Coimmunoprecipitation Assay

Rat cortical tissue and cell proteins were extracted using RIPA Lysis Buffer (Beyotime, Shanghai, China) supplemented with protease inhibitor cocktail (Roche, IN, USA). Proteins were quantified with a BCA kit (Beyotime, Shanghai, China); equivalent amounts of proteins (30 µg) were electrophoresed on 12% SDS-PAGE, followed by transferring onto 0.2 µm pore-size polyvinylidene fluoride (PVDF) membranes (Millipore, Billerica, MA, USA). After blocking with 5% non-fat milk solution for 1 h at room temperature, the membranes were washed with TBS-Tween-20 (0.1%, *v*/*v*). The membranes were incubated with corresponding primary antibodies at 4 °C overnight and then incubated with HRP-conjugated secondary antibodies (Bioworld, St. Louis Park, MN, USA) for 1 h at room temperature. The antibody-antigen complexes were visualized by the chemiluminescence method using the enhanced ECL Immunoblotting System (Bio Tanon, Shanghai, China).

For coimmunoprecipitation, cell pellets were lysed with ice-cold lysis buffer, and lysates were centrifuged (12,500× *g*) at 4 °C for 15 min. Proteins were immunoprecipitated with 5 µg Nrf2 antibody at 4 °C overnight. The immunocomplexes were incubated with precleared protein A/G-agarose beads (Santa Cruze Biotechnology, Dallas, TX, USA) at 4 °C for 2 h and were washed with the lysis buffer. Samples were then subjected to immunoblotting.

### 2.7. Quantitative Real-Time PCR (qRT-PCR)

Acyl-CoA synthetase long-chain family member 4 (ACSL4), GPX4, ferritin heavy chain (FTH1), transferrin (TF), solute carrier family 7 member 11 (SLC7A11) and GSS expression were measured using quantitative real-time PCR. Total RNA was extracted using TRIZOL reagent (Thermo Fisher Scientific, MA, USA). RNA concentrations were assessed using a NanoDrop 2000 Spectrophotometer (Thermo Fisher Scientific, USA), and then 1 µg total RNA was reverse-transcribed to cDNA using a HiScript II RT reagent kit (Vazyme, Nanjing, Jiangsu, China) according to the manufacture’s protocol. Aliquots of 10 ng cDNA were used as templates for real-time PCR reactions. qRT-PCR was performed using AceQ qPCR SYBR Green Master Mix (Vazyme, Nanjing, China) and was run on the Step One Plus Real Time PCR system (Applied Biosystems, Foster City, CA, USA). qRT-PCR ran for 40 cycles with a Tm of 60 °C. Relative gene expression was calculated using the 2^−ΔΔCt^ method [36] and was normalized to β-actin. All primer sequences are listed in Supplementary Table S1.

### 2.8. Fe^2+^ Level Measurement

Intracellular Fe^2+^ levels were measured using an Iron Assay Kit (Abcam, Cambridge, MA, USA) according to the manufacturer’s instructions. Rat cortical tissues or cells were homogenized in iron assay buffer and centrifuged at 16,000× *g* for 10 min. To detect Fe^2+^ levels, an iron probe containing an iron chromogen Ferene S was added to each sample. Samples were incubated with the iron probe at 37 °C for 60 min. The absorbance was measured using a microplate reader (Tecan, Crailsheim, Germany) at 593 nm.

### 2.9. Measurement of GSH, MDA and H_2_O_2_ Levels

Rat cortical tissues of ipsilateral hemispheres were homogenized, and cells were lysed. Samples were centrifuged at 10,000× *g* for 10 min, and the supernatants were collected. GSH and GSSG contents in the supernatant of tissues and cells were detected using the GSH and GSSG Assay Kit (Beyotime, Shanghai, China) according to the manufacturer’s protocol. To measure the Malondialdehyde (MDA) and H_2_O_2_ levels, rat cortical tissues or cells were homogenized or lysed, respectively. Samples were centrifuged at 12,000× *g* for 10 min, and the supernatants were collected. MDA and H_2_O_2_ levels in the supernatants were detected by using MDA Assay Kit (Beyotime, Shanghai, China) and H_2_O_2_ Assay Kit (Beyotime, Shanghai, China), respectively, according to the manufacturer’s instructions.

### 2.10. GPX Activity Determination

Rat cortical tissues and cells were homogenized or lysed, respectively, and then were centrifuged at 12,000 rpm for 10 min. The supernatants were subjected to measurement of glutathione peroxidase (GPX) activity with the GPX Assay Kit (Beyotime, Shanghai, China) according to the manufacturer’s instructions. In brief, samples were incubated with a solution containing 1 mM GSH, 0.2 mM NADPH and 0.4 U/mL glutathione reductase for 15 min at room temperature. A total of 0.22 mM tert-butyl hydroperoxide was added to samples to initiate the reaction. The rate of decrease in absorption of NADPH at 340 nm was measured. GPX activity was defined based on the consumption of NADPH per minute per milligram of protein. NADPH consumption was calculated by using a millimolar extinction coefficient for an NADPH of 6.22.

### 2.11. Statistical Analysis

All data are presented as mean ± SD. Statistical analyses were performed using GraphPad Prism 8.0 (GraphPad). Statistical significance was determined using one-way ANOVA followed by Turkey’s post hoc test or two-way ANOVA with the Bonferroni post hoc test for multiple comparisons; *p* < 0.05 denotes statistical significance.

## 3. Results

### 3.1. γ-GC Protects against MCAO/R-Induced Neuron Death

**Figure 1:**
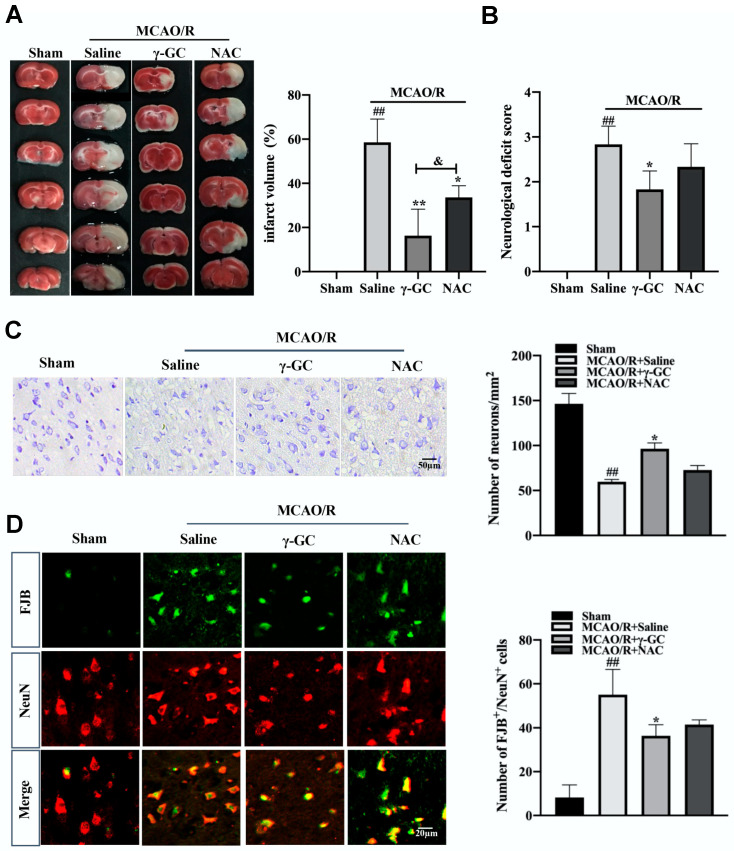
γ-GC inhibits MCAO/R-induced neuron death. Rats were treated with γ-GC (688 mg/kg body weight) and NAC (522 mg/kg body weight) 1.5 h after being subjected to MCAO. Twenty-four hours after reperfusion, the following observations were performed. (**A**) The brain sections were subjected to TTC staining to measure the infarction volume. (**B**) Neurological deficits of rats were evaluated. (**C**) Paraffin sections of rat cerebral cortex were stained with Nissl staining solution, and then Nissl positive cells were counted (Scale bar, 50 µm). (**D**) Paraffin sections of the rat cerebral cortex were co-stained with FJB (Green) and NeuN (Red) (Scale bar, 20 µm), and the FJB^+^/NeuN^+^ cells were counted. Data are mean ± SD (n = 6, 6 rats/group), ## *p* < 0.01 versus Sham group, * *p* < 0.05 and ** *p* < 0.01 versus MCAO/R + saline group, & *p* < 0.05 versus MCAO/R + γ-GC group.

Neuron damage or death is the main phenomenon in CIR-induced brain injury [37,38]. The rat MCAO/R model was used to observe the effect of γ-GC on neuron death in the rat brain after cerebral ischemia/reperfusion. Rats were orally administrated with γ-GC (688 mg/kg body weight) 1.5 h after MCAO was performed. Twenty-four hours after reperfusion, the rat brains were subjected to TTC staining. As shown in Figure 1A, there was no cerebral infarction in Sham rats, whereas the infarction volume in rats of the MCAO/R group increased. γ-GC significantly reduced the infarction volume in the affected brain area. Although NAC also decreased the infarction volume, it showed weaker efficiency than γ-GC. Consistently, γ-GC apparently reduced neurological deficit scores, suggesting that γ-GC alleviated CIR-induced neurological dysfunction (Figure 1B). Since CIR-induced brain injury is related with neuron damage, we next observed if γ-GC could affect cerebral neuron death induced by MCAO/R. Nissl staining (see Supplementary Methods) showed that the number of neurons in the MCAO/R-affected cortex of the ipsilateral hemisphere decreased, suggesting neuron loss after MCAO/R. Compared with saline-treated MCAO/R rats, γ-GC but not NAC treated MCAO/R rats showed more Nissl-positive cells in the brains (Figure 1C). On the other hand, the number of FJB-positive neurons was markedly increased in the rat cortex after MCAO/R, whereas γ-GC administration reduced the number of positive neurons, and the effect of γ-GC was better than NAC (Figure 1D). Taken together, the above results indicate that γ-GC effectively inhibited MCAO/R-induced neuron death.

### 3.2. γ-GC Inhibits MCAO/R-Induced Neuronal Ferroptosis

**Figure 2:**
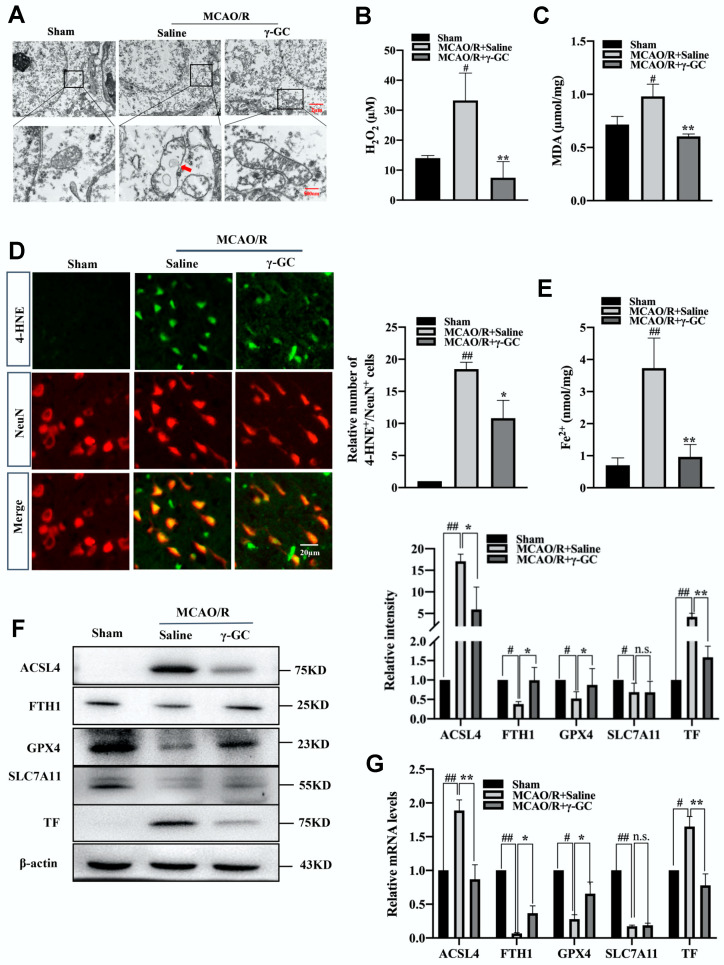
γ-GC inhibits MCAO/R-induced neuron ferroptosis. Rats were treated with γ-GC (688 mg/kg body weight) 1.5 h after being subjected to MCAO. Twenty-four hours after reperfusion, the following observations were performed. (**A**) The ultrastructure of neurons in the cortex was detected by transmission electron microscopy (Scale bar, 2 µm and 500 nm). (**B**,**C**) H_2_O_2_ and MDA contents in cortex tissues were determined using the commercial detecting kits. (**D**) Paraffin sections of rat cerebral cortex were immunofluorescence stained with 4-HNE (Green) and NeuN (Red), and the 4-HNE/NeuN double-stained cells in the cortex were quantitatively analyzed (Scale bar, 20 µm). (**E**) Fe^2+^ content in cortex tissue was tested using an iron assay kit. (**F**,**G**) The protein and mRNA levels of the indicated protein in cortex tissues were detected by immunoblotting and qRT-PCR assay. Data are mean ± SD (n = 3, 3 rats/group). # *p* < 0.05 and ## *p* < 0.01 versus Sham group; * *p* < 0.05 and ** *p* < 0.01 versus MCAO/R + saline group.

Previous reports show that ferroptosis is involved in cerebral ischemia/reperfusion injury [18,19]. We investigated whether γ-GC affected neuronal ferroptosis. A typical ferroptosis characteristic is the injury of mitochondrion [39]. As shown in Figure 2A, MCAO/R resulted in the injuries of cerebral neuron mitochondrial morphology, including the reducing or vanishing of mitochondria crista and rupturing of the outer membrane (see Supplementary Methods). Interestingly, these mitochondrial injuries were significantly alleviated by γ-GC treatment. We next proceeded to explore the effect of γ-GC on the accumulation of excessive lipid peroxidation, a hallmark of ferroptosis [40]. In neurons, Fe^2+^ could convert H_2_O_2_ into the most active hydroxyl radical through Fenton reaction, thus facilitating lipid peroxidation of cell membrane and leading to ferroptosis [41,42]. In the present study, γ-GC significantly reduced not only the cerebral H_2_O_2_ level after MCAO/R but also inhibited the MCAO/R-induced increase of MDA, an end-product of lipid peroxidation (Figure 2B,C). Further immunofluorescence staining showed that γ-GC suppressed MCAO/R-induced increase of 4-hydroxy-2-nonenol (4-HNE) in cerebral neurons (Figure 2D). The key reason for ferroptosis is the abnormal accumulation of reactive free iron (Fe^2+^) in cells. As shown in Figure 2E, the MCAO/R-upregulated cerebral Fe^2+^ level was apparently inhibited by γ-GC. Next, we examined the mRNA and protein levels of ferroptosis related protein by qPCR and immunoblotting assays. Results from the qPCR assay indicated that MCAO/R led to the reduction of cerebral FTH1, GPX4 and SLC7A11 mRNA levels and the increase of ACSL4 and TF mRNA levels. γ-GC reversed MCAO/R-induced alterations of FTH1, GPX4, ACSL4 and TF but not SLC7A11 both in mRNA and protein levels (Figure 2G,F). Further immunofluorescence analyses demonstrated that γ-GC inhibited MCAO/R-induced changes of protein levels of FTH1, GPX4, ACSL4 and TF but not SLC7A11 in cortical neurons (Figure S1). Overall, these findings demonstrated that γ-GC effectively inhibited MCAO/R-induced neuronal ferroptosis.

### 3.3. γ-GC Increases the GSH Level in Neurons In Vivo and In Vitro

**Figure 3:**
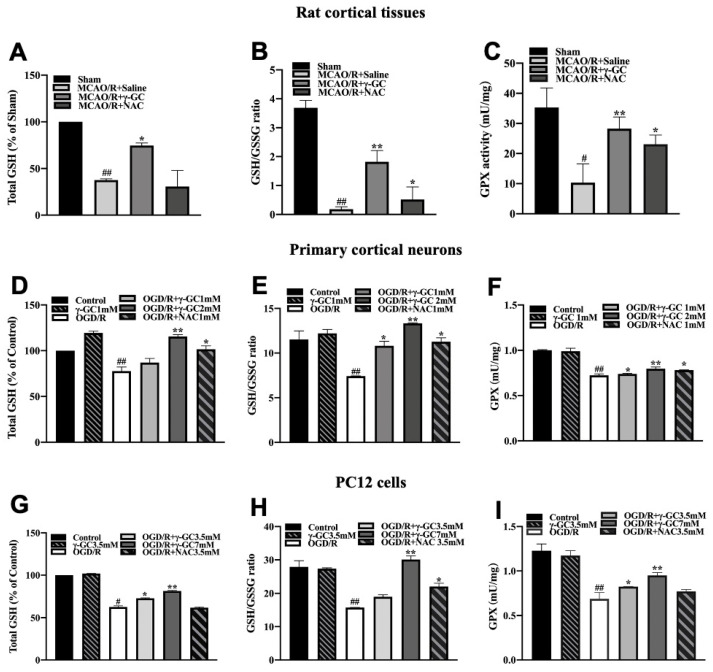
γ-GC increases the level of neuronal GSH. (**A**–**C**) Rats were administrated with γ-GC (688 mg/kg body weight) 1.5 h after being subjected to MCAO. Twenty-four hours after reperfusion, (**A**) GSH content, (**B**) GSH/GSSG ratio and (**C**) GPX enzyme activity in cortical tissue were determined (n = 6, 6 rats/group). (**D**–**F**) Primary cortical neurons were treated with indicated concentration of γ-GC after OGD (n = 3). Twelve hours after reoxygenation, (**D**) GSH content, (**E**) GSH/GSSG ratio and (**F**) GPX enzyme activity were determined. (**G**–**I**) PC12 cells were treated with indicated concentration of γ-GC after OGD (n = 3). Twelve hours after reoxygenation, (**G**) GSH content, (**F**) GSH/GSSG ratio and (**I**) GPX enzyme activity were determined. Data are mean ± SD, # *p* < 0.05 and ## *p* < 0.01 versus Sham group or control cells; * *p* < 0.05 and ** *p* < 0.01 versus MCAO/R group or OGD/R treated cells.

In cerebral stroke, the decrement of GSH levels in the brain may aggravate oxidative stress [43]. GSH is a cofactor of GPX4 that can detoxify lipid peroxidation and inhibit ferroptosis [44]. As the precursor dipeptide of GSH, exogenous γ-GC supplement might increase cellular GSH level. As expected, γ-GC but not NAC significantly upregulated the GSH level and GSH/GSSG ratio and increased GPX enzyme activity in the cerebral cortex after MCAO/R (Figure 3A–C). We then treated primary neurons with 0.25, 0.5, 1 or 2 mM γ-GC and treated PC12 cells with 0.85, 1.7, 3.5 or 7 mM γ-GC after cells were exposed to OGD/R. The reduction of cell viability induced by OGD/R was inhibited by 0.5, 1 and 2 mM γ-GC in primary neurons and 3.5 and 7 mM γ-GC in PC12 cells (Figure S2). Next, we examined the effect of γ-GC on GSH levels after cells were exposed to OGD/R. The results showed that γ-GC significantly upregulated GSH levels and the GSH/GSSG ratio and GPX activity in primary neurons (Figure 3D–F) and PC12 cells (Figure 3G–I) after OGD/R, respectively. Collectively, the above results indicate that γ-GC increased neuronal GSH levels, suggesting that γ-GC could enhance the enzyme activity of GPX4 and as a result inhibit neuronal ferroptosis.

### 3.4. γ-GC Increases GSS Expression In Vivo and In Vitro

**Figure 4:**
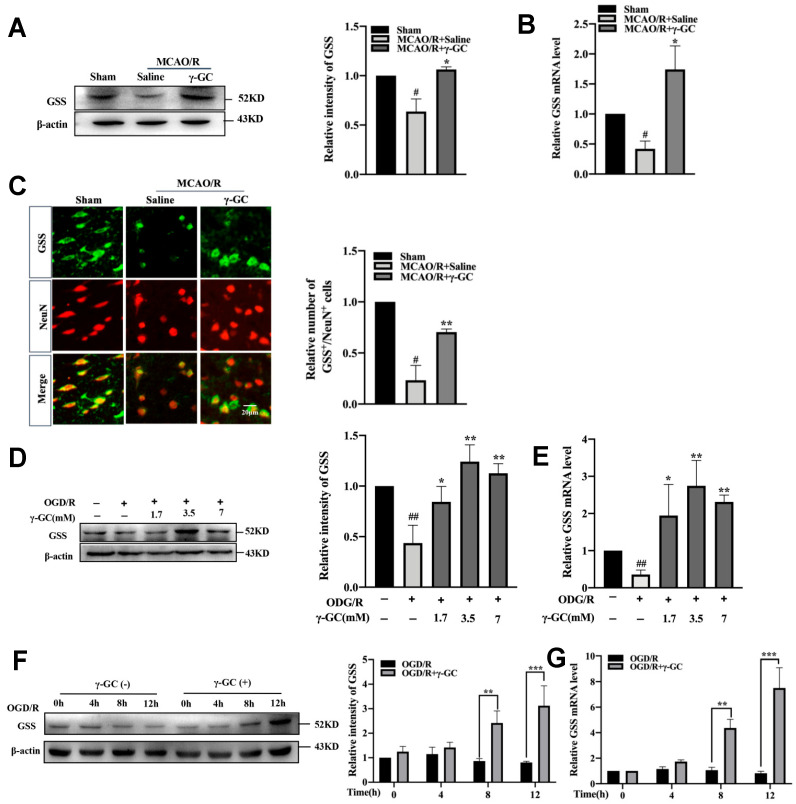
γ-GC increases neuronal GSS expression in vivo and in vitro. (**A**–**C**) Rats were treated with γ-GC (688 mg/kg body weight) 1.5 h after being subjected to MCAO (n = 3, 3 rats/group). Twenty-four hours after reperfusion, the following observations were performed. (**A**,**B**) The mRNA and protein levels of GSS were detected by qRT-PCR and immunoblotting assays, respectively. (**C**) Paraffin sections of the rat cerebral cortex were co-immunostained with GSS and NeuN antibodies, and the numbers of GSS^+^/NeuN^+^ cells in the cortex were quantitatively analyzed (Scale bar, 20 µm). PC12 cells were treated with OGD for 4 h (n = 3), and γ-GC (1.7, 3.5 and 7 mM) was added into the cell culture at the onset of reoxygenation. (**D**,**E**) Twelve hours after reoxygenation, the mRNA and protein levels of GSS were detected by qPCR and immunoblotting. (**F**,**G**) PC12 cells were treated with γ-GC (3.5 mM) for the indicated time after reoxygenation, and then mRNA and protein levels of GSS were detected by qRT-PCR and immunoblotting assays, respectively. Data are mean ± SD, # *p* < 0.05 and ## *p* < 0.01 versus Sham group or control cells; * *p* < 0.05, ** *p* < 0.01 and *** *p* < 0.001 versus MCAO/R group or OGD/R cells.

GSS is the enzyme for GSH synthesis from γ-GC [21]. We found that the mRNA and protein levels of GSS significantly decreased in the cortical tissue after MCAO/R (Figure 4A,B). Importantly, γ-GC significantly inhibited MCAO/R-induced reduction of cortical GSS mRNA and protein levels (Figure 4A,B). The immunofluorescence result demonstrated that the MCAO/R-induced decrease of GSS/NeuN positive cell numbers was inhibited by administration of γ-GC, suggesting γ-GC promoted GSS expression in neurons (Figure 4C). Further in vitro study was performed to evaluate the effect of γ-GC on GSS expression in detail. We treated PC12 cells with 1.7, 3.5 and 7 mM γ-GC after cells were exposed to OGD/R. Results from qRT-PCR and immunoblotting showed that γ-GC significantly elevated GSS mRNA and protein levels, and γ-GC at concentration of 3.5 mM showed the strongest effect (Figure 4D,E). As shown in Figure 4F,G, 3.5 mM γ-GC time-dependently elevated GSS mRNA and protein levels in PC12 cells during 8 h of observation after cells were subjected to OGD/R. Overall, both in vivo and in vitro studies indicate that γ-GC increases neuronal GSS expression after MCAO/R and OGD/R, suggesting that γ-GC increases GSH not only by acting as the precursor dipeptide of GSH but also by promoting GSS expression.

### 3.5. γ-GC Activates Neuronal Nrf2 In Vivo and In Vitro

**Figure 5:**
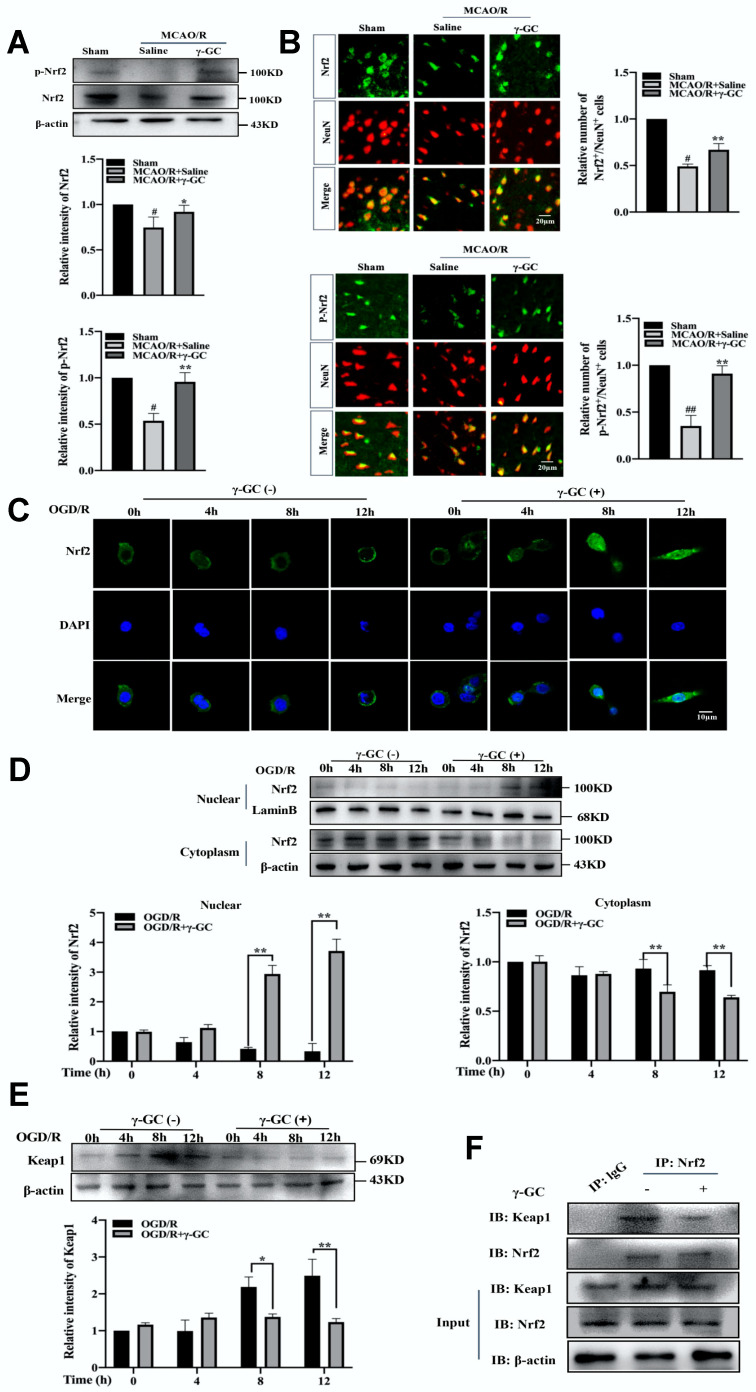
γ-GC activates neuronal Nrf2. (**A**,**B**) Rats were treated with γ-GC (688 mg/kg body weight) 1.5 h after being subjected to MCAO (n = 3, 3 rats/group). Twenty-four hours after reperfusion, (**A**) the total and phosphorylated Nrf2 protein levels were detected by immunoblotting and (**B**) the numbers of phosphorylated Nrf2 (p-Nrf2)/NeuN and Nrf2/NeuN positive cells were observed using fluorescence-microscopy (n = 3). (**C**–**E**) PC12 cells were treated with OGD for 4 h (n = 3). γ-GC was added into the cell culture at the onset of reoxygenation. Twelve hours after reoxygenation, (**C**) Nrf2 (Green) distribution was observed with confocal scanning microscopy (the nucleus was stained with DAPI), (**D**) protein levels of Nrf2 in cytoplasmic and nuclear fractions of PC12 cells were determined by immunoblotting, and (**E**) the protein level of Keap1 was detected by immunoblotting. (**F**) Co-immunoprecipitation assay was used to test the interaction of endogenous Nrf2 with Keap1. Data are mean ± SD, # *p* < 0.05, ## *p* < 0.01 versus Sham group; * *p* < 0.05 and ** *p* < 0.01 versus MCAO/R group or the group indicated.

Nrf2, a master transcription factor, is responsible for maintaining redox homeostasis in ischemic stroke [45]. In the present study, we found that γ-GC increased the total Nrf2 protein and phosphorylated Nrf2 protein levels after MCAO/R (Figure 5A). Consistently, immunofluorescence assay also showed that γ-GC increased the total Nrf2 protein and phosphorylated Nrf2 protein levels in cortical neurons (Figure 5B). We then observed the cytoplasmic and nuclear distribution of Nrf2 in PC12 cells by using confocal microscopy. As shown in Figure 5C, γ-GC facilitated nuclear translocation of Nrf2 in OGD/R-exposed PC12 cells 8 h after reoxygenation. Immunoblotting assay also showed that γ-GC increased nuclear Nrf2 and reduced cytosolic Nrf2 in OGD/R-exposed PC12 cells 8 h after reoxygenation (Figure 5D; see Supplementary Methods). Keap1, a repressor of the Nrf2 pathway, plays a key role in regulating Nrf2 activity [46]. The immunoblotting assay showed that γ-GC suppressed the ODG/R-induced increase of Keap1 protein level 8 h after reoxygenation (Figure 5E). Results from co-immunoprecipitation assay showed that γ-GC facilitated the dissociation of Nrf2 from Keap1 (Figure 5F). These data strongly suggest that γ-GC activates Nrf2 and promotes its nuclear translocation during cerebral ischemia/reperfusion.

### 3.6. γ-GC Increases GSS Expression through Activating Nrf2

**Figure 6:**
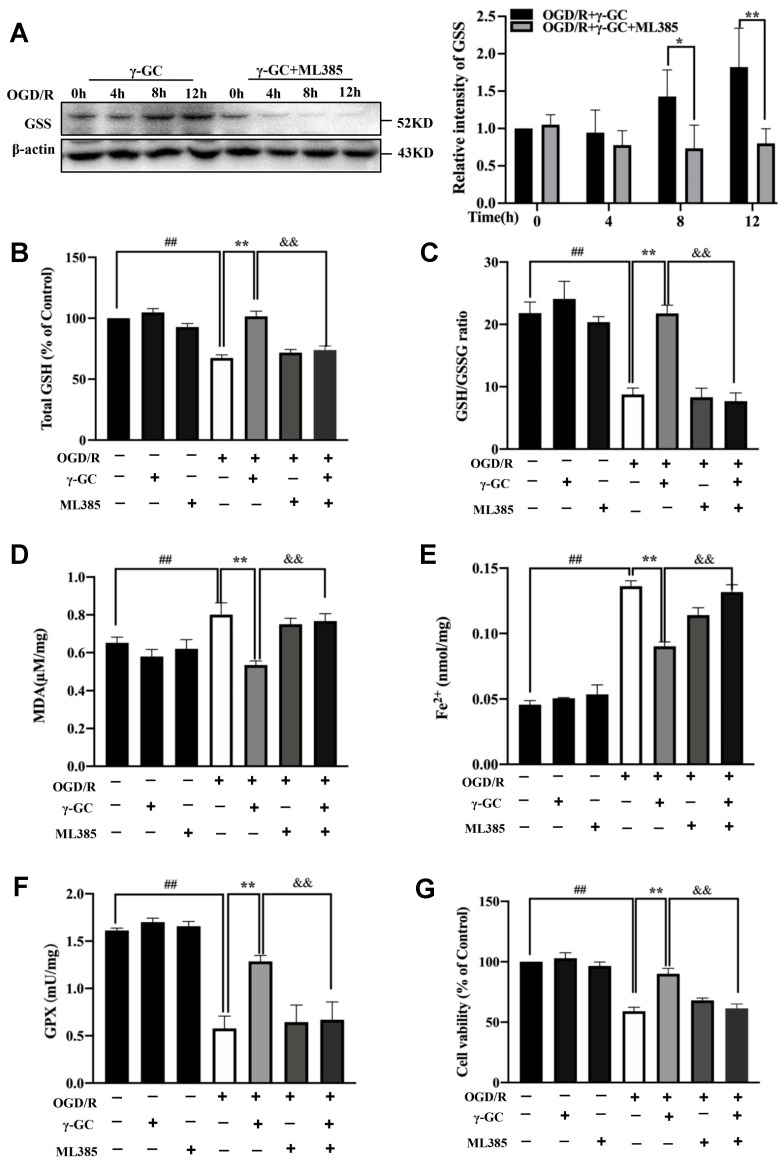
γ-GC increases the protein level of GSS and GSH contents in PC12 cells through activating Nrf2. PC12 cells were pretreated with Nrf2 inhibitor ML385 (2 µM) 2 h before OGD and then were treated with γ-GC (3.5 mM) at the onset of reoxygenation for the indicated time. (**A**) Immunoblotting was performed to measure the protein level of GSS. (**B**,**C**) Intracellular GSH content and the GSH/GSSG ratio were determined. (**D**–**G**) The MDA and Fe^2+^ levels, GPX enzyme activity and cell viability were detected using commercial detection kits. Data are mean ± SD (n = 3), ## *p* < 0.01 versus control group, * *p* < 0.05 and ** *p* < 0.01 versus OGD/R cells or the group indicated, && *p* < 0.01 versus OGD/R+γ-GC cells.

The above results show that γ-GC promoted phosphorylation and nuclear translocation of Nrf2. We next explored if the activation of Nrf2 is important for the effect of γ-GC on GSS expression. PC12 cells were pretreated with Nrf2 inhibitor ML385 (2 µM) and then were subjected to OGD/R followed by γ-GC (3.5 mM) treatment. The result from immunoblotting showed that ML385 significantly suppressed γ-GC-induced elevation of GSS protein level in OGD/R-treated PC12 cells (Figure 6A). Consistently, the γ-GC-induced increase of GSH in PC12 cells was also inhibited by ML385 (Figure 6B,C). Since the level of GSH in cells is the key factor for ferroptosis inhibition, ML385 reversed the effects of γ-GC in decreasing MDA and Fe^2+^ deposits and enhancing GPX activity and cell viability after OGD/R (Figure 6D–G). Collectively, these results indicate that γ-GC protects neuronal from ferroptosis by activating Nrf2 to increase GSS expression and GSH levels.

### 3.7. γ-GC Activates Nrf2 through Promoting Phosphorylation of PKC-ε

**Figure 7:**
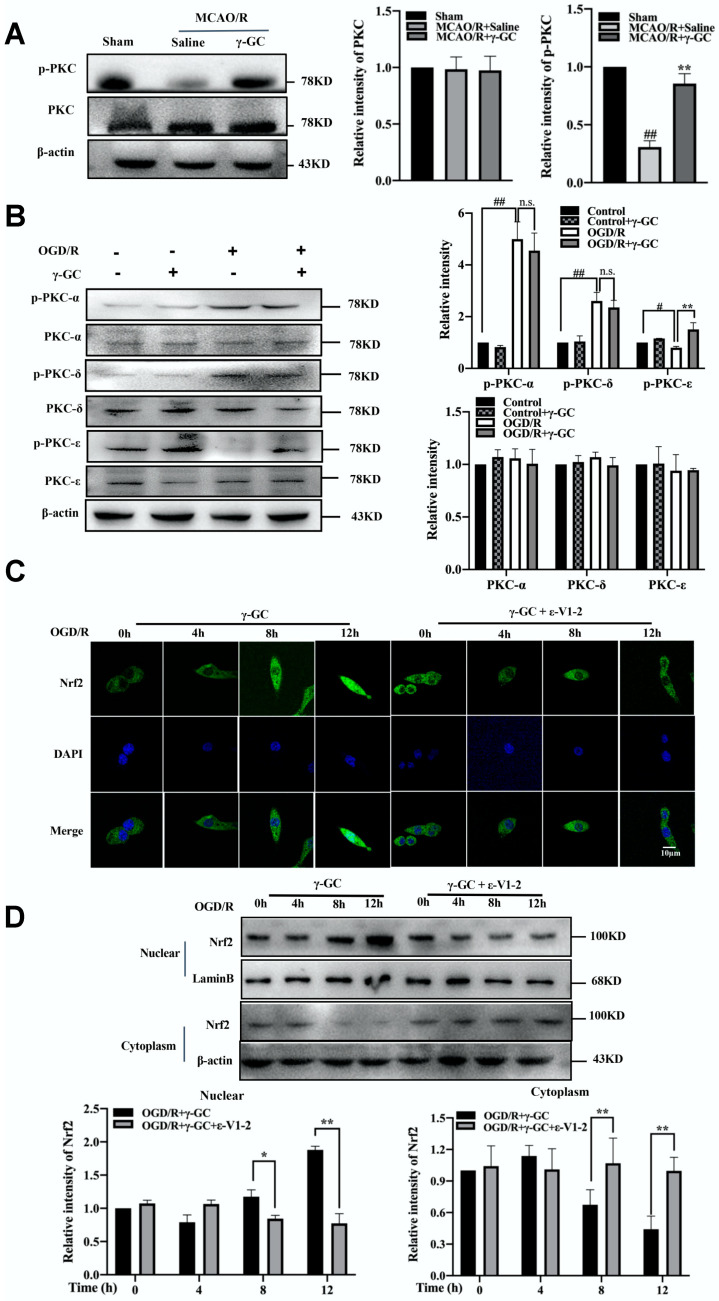
γ-GC activates Nrf2 through promoting phosphorylation of PKC-ε. (**A**) Rats were treated with γ-GC (688 mg/kg body weight) after being subjected to 1.5 h of MCAO. Twenty-four hours after reperfusion, ischemia cerebral cortical tissues were subjected to immunoblotting by using PKC and p-PKC antibodies (n = 3, 3 rats/group). (**B**) PC12 cells were treated with OGD for 4 h (n = 3). γ-GC was added into cell culture at the onset of reoxygenation. Twelve hours after reoxygenation, the cell lysates were subjected to immunoblotting by using PKC-α, PKC-δ, PKC-ε, p-PKC-α, p-PKC-δ and PKC-ε antibodies (n = 3). (**C**,**D**) PC12 cells were pretreated with PKC-ε inhibitor ε-V1-2 (20 µM) 2 h before OGD and then were treated with γ-GC at the onset of reoxygenation, and after the indicated time for reoxygenation, cells were subjected to confocal scanning microscopy to detect the distribution of Nrf2 (n = 3, (**C**)), and the nuclear and cytoplasmic fractions of protein were subjected to immunoblotting by using Nrf2 antibody (n = 3, (**D**)). Data are mean ± SD, # *p* < 0.05 and ## *p* < 0.01 versus Sham group or indicated group; * *p* < 0.05 and ** *p* < 0.01 versus MCAO/R group or indicated group. n.s.:not significant.

We next explored the underlying mechanism by which γ-GC activated Nrf2. PKC, a ubiquitous protein kinase, is upstream Nrf2 [47]. Hence, we observed if γ-GC activated Nrf2 through phosphorylating PKC. As shown in Figure 7A, γ-GC significantly increased the phosphorylated protein level of PKC in the cerebral cortex after MCAO/R. Further immunofluorescence observation showed that γ-GC apparently increased the level of phosphorylated PKC in cerebral cortical neurons after MCAO/R (Figure S3). There are various PKC isoforms in the brain, among which PKC-α, PKC-δ and PKC-ε are involved in ischemic injury [48,49]. We thus detected the effects of γ-GC on the phosphorylation of PKC-α, PKC-δ and PKC-ε in OGD/R-treated PC12 cells. The results showed that OGD/R did not alter the total levels of PKC-α, PKC-δ and PKC-ε, but elevated phosphorylated PKC-α and PKC-δ levels and reduced phosphorylated PKC-ε levels (Figure 7B). We noted that γ-GC reversed OGD/R-induced reduction of PKC-ε phosphorylation but did not affect phosphorylation of PKC-α and PKC-δ (Figure 7B). To observe if γ-GC activated Nrf2 by phosphorylating PKC-ε, we pretreated PC12 cells with PKC-ε inhibitor ε-V1-2 and then detected the effect of γ-GC on Nrf2 activation during OGD/R. The results from confocal microscopy and immunoblotting assay showed that after ε-V1-2 treatment, γ-GC failed to promote nuclear translocation of Nrf2 (Figure 7C,D), suggesting that γ-GC activated Nrf2 in PC12 through phosphorylating PKC-ε during OGD/R.

## 4. Discussion

Cerebral ischemia/reperfusion injury is an inevitable secondary injury caused by vessel recanalization, which may exacerbate damage and dysfunction of the ischemic brain area [50,51]. During cerebral ischemia/reperfusion, massive O_2_ arrives and produces large amounts of ROS [10]. Because of high metabolic rates of oxygen consumption and high levels of PUFAs, neurons are known to be more susceptible to oxidative stress than other organs [11]. Increasing evidence demonstrates that ischemic stroke causes the rapid depletion of cellular GSH [52,53,54]. Ferroptosis is a recently described form of cell death driven by iron-dependent lipid peroxidation [55]. Recently, studies have shown that iron overload aggravates ischemic insult induced by focal ischemia and early reperfusion, and inhibiting ferroptosis could alleviate CIR-induced brain injury [18,56]. In the present study, γ-GC significantly decreased infarct volume and neurological deficit scores in MCAO/R rats. Administration of γ-GC at 1.5 h after MCAO is effective for inhibiting cerebral ischemia/reperfusion-induced oxidative stress and brain tissue injury. The development of cerebral ischemia/reperfusion injury is different between humans and rats; thus, the γ-GC administration is also different. Consistently, γ-GC inhibited cortical neuron loss and death occurred in the ischemic hemisphere. We noted that the neuroprotective effect of γ-GC is better than NAC. It is not surprising that NAC is a cysteine prodrug for synthesis of GSH through two-step enzyme catalyzed reaction, and it appears to be an effective drug only for decreasing the toxicity of acetaminophen overdose [25]. Γ-GC, as an precursor for GSH synthesis, readily penetrated the BBB and immediately synthesized GSH just catalyzed by GSS [28].

Our study first found that γ-GC alleviates CIR-induced neuronal ferroptosis. As the main driving force of ferroptosis lipid peroxidation induces the instability of the mitochondrial membrane [55], mitochondrial morphology alterations display typical characteristics of ferroptosis, including reduction or vanishing of mitochondria crista and rupturing of the outer membrane [39]. In the present study, these ferroptosis-related mitochondrial morphology alterations in MCAO/R rats were significantly inhibited by γ-GC. H_2_O_2_ exacerbates lipid peroxidation via Fenton reaction [41], and both MDA and 4-HNE are major end-products of lipid peroxidation [57]. The MDA and 4-HNE levels are related with the extent of cerebral ischemia/reperfusion injury and ferroptosis [58]. As expected, we found that γ-GC treatment not only increased GSH in the brain of MCAO/R rats but also suppressed MCAO/R-induced increments of H_2_O_2_, 4-HNE and MDA levels in rat cortical tissue after MCAO/R. Our study also demonstrated that γ-GC remarkably inhibited the MCAO/R-induced iron depositions in the cerebral cortex.

Ferroptosis is a complex process regulated by various proteins. ACSL4 is a key enzyme that regulates lipid composition and contributes to ferroptosis [59]. SLC7A11 is the *cystine*/glutamate transporter involved in GSH synthesis [60]. As the important negative regulator of ferroptosis, GPX4 utilizes reduced GSH as a cofactor to reduce lipid hydroperoxides to lipid alcohols and thus mitigates lipid peroxidation [14]. TF promotes cellular iron uptake, but FTH1 is a ubiquitous intracellular protein that stores iron in a soluble, non-toxic and readily available form [61]. TF and FTH1 play the opposite role in ferroptosis development. Therefore, it was not surprising that in MCAO/R rats, the expression of ACSL4 and TF increased, and the expression of SLC7A11, GPX4 and FTH1 in the cerebral cortex decreased. We found that γ-GC significantly suppressed MCAO/R-induced changes of ACSL4, TF, GPX4 and FTH1 but not SLC7A11. Among these proteins, GPX4 is the key regulator for inhibiting ferroptosis. Since GSH acts as the reducing substrate of GPX4, the neuronal GSH is indispensable for mitigating lipid peroxidation and inhibiting ferroptosis [62]. GSH depletion directly suppresses GPX4 activity and triggers lipid peroxidation [63]. Cerebral ischemia/reperfusion may cause the rapid depletion of cellular GSH in brains [52,53,54]. As the brain presents a low ability to synthesize and regenerate GSH [64], to increase GSH is important for enhancing the activity of GPX4 and inhibiting ferroptotic neuron death.

Exogenous GSH supplementation shows no significant effect due to its poor bioavailability [65,66]. As noted, γ-GC has been reported to act as an effective and safe agent for augmentation of GSH [67]. In the present study, both our in vivo and in vitro studies indicated that γ-GC remarkably increased the GSH level, which was reduced in the rat MCAO/R and cell OGD/R models. GSS is critical enzyme for GSH synthesis and catalyzes the addition of L-glycine to γ-GC to form GSH [68]. GSS deficiency results in cell membrane rupture due to the lack of GSH [69]. In the present study, we found that MCAO/R down-regulated rat cortical GSS, which suggested that if γ-GC acted merely as the raw material for GSH synthesis, it might not increase GSH level apparently. Interestingly, the results of the present study indicated that exogenous γ-GC significantly enhances the expression of GSS. This finding displayed a novel function of γ-GC, as the precursor γ-GC also worked as a regulator of the GSH synthase system to raise GSH levels.

Transcription factor Nrf2 induces the expression of cytoprotective and detoxification genes [70]. It has been reported that Nrf2 could drive the expression of GSS and enhance the level of reduced GSH [71]. As expected, γ-GC increased the total protein level and phosphorylation of Nrf2 after MCAO/R in vivo and OGD/R in vitro. Normally Nrf2 combines with Keap1, which retains Nrf2 in the cytoplasm. Γ-GC down-regulated Keap1 to reduce the combination between Nrf2 with Keap1 and resulted in Nrf2 translocating from cytoplasm into the nucleus. PKC is upstream of Nrf2, and phosphorylated PKC may activate Nrf2 [45]. In the present research, we found that γ-GC enhanced the phosphorylation of PKC in the rat cortical cortex after MCAO/R. Previous studies have reported that PKC-α, PKC-δ and PKC-ε are involved in ischemic injury [48,49]. We found that γ-GC promoted phosphorylation of PKC-ε but not PKC-α and PKC-δ. After PKC-ε inhibitor treatment, the effects of γ-GC in promoting the phosphorylation and nuclear translocation of Nrf2 were suppressed, suggesting that γ-GC actives Nrf2 through phosphorylating PKC-ε. To evaluate whether γ-GC enhanced GSS expression, we used specific Nrf2 inhibitor ML385 to pretreat neuronal cells before OGD/R. As expected, ML385 apparently reversed γ-GC-induced enhancement of GSS expression and increased GSH levels in OGD/R cells. ML385 also reversed the γ-GC-induced an increase of cell viability and GPX activity and the reduction of MDA and Fe^2+^ levels in OGD/R cells. These results confirmed that γ-GC inhibited neuronal ferroptosis in cerebral ischemia/reperfusion through activating Nrf2 to enhance GSS expression and upregulate GSH levels. The mechanism in detail is that γ-GC not only acts as a raw material replenishing intracellular GSH but also promotes GSH synthesis by increasing GSS expression via regulating the PKC-ε/Nrf2 pathway.

## 5. Conclusions

Taken together, this study demonstrates that γ-GC attenuates cerebral ischemia/reperfusion injury through inhibiting neuronal lipid peroxidation and ferroptosis. The most important finding in the present study is that, as the precursor of GSH, γ-GC also promotes the expression of GSS for GSH synthesis. The mechanism analysis indicates that γ-GC upregulates GSS via activating the PKC-ε/Nrf2 pathway. Our research provides a novel strategy for the treatment of cerebral ischemia/reperfusion.

## Data Availability

The data are contained within the article or supplementary materials.

## Supplementary Materials

The following supporting information can be downloaded at: https://www.mdpi.com/article/10.3390/antiox11091653/s1, Materials and methods includes: Cell nuclear and cytoplasm protein extraction, Nissl staining, Transmission electron microscopy analysis and TTC staining; Figure S1: Effects of γ-GC on ferroptosis related protein levels after MCAO/R; Figure S2: Effects of γ-GC on neuronal cell viability; Figure S3: γ-GC increased the level of phosphorylated PKC in cortical neurons after MCAO/R; Table S1: Primer sequences for qPCR.
