# Supplementary material for: γ-Glutamylcysteine Exerts Neuroprotection Effects against Cerebral Ischemia/Reperfusion Injury through Inhibiting Lipid Peroxidation and Ferroptosis
Source: Antioxidants (Basel). 2022 Aug 25;11(9):1653. doi: 10.3390/antiox11091653 (PMC9495808; doi:10.3390/antiox11091653)
Supplement: Supplementary file 1 [file antioxidants-11-01653-s001.zip › antioxidants-1877720-supplementary.pdf]

γ-glutamylcysteine exerts neuroprotection effects against cerebral ischemia/reperfusion injury through inhibiting lipid peroxidation and ferroptosis

Ruyi Zhang [1], Jianzhen Lei [1], Luyao Chen [1], Yanan Wang [1], Guocui Yang [1], Zhimin Yin [2,*] and Lan Luo [1,*]

1 State Key Laboratory of Pharmaceutical Biotechnology, School of Life Sciences, Nanjing University, Nanjing 210023, Jiangsu, China
2 Jiangsu Province Key Laboratory for Molecular and Medical Biotechnology, College of Life Science, Nanjing Normal University, Nanjing 210046, Jiangsu, China
* Correspondence: lanluo@nju.edu.cn (L.L.); Tel/fax: +86 25 89682705. yinzhimin@njnu.edu.cn(Z.Y.); Tel/Fax:+86 25 85891305

Materials and methods

Antibodies

All the antibodies used are commercially validated antibodies. Primary antibodies for immunoblotting were as follows: anti-ACSL4, anti-FTH1, anti-p-PKC-δ, anti-p-PKC-α and anti-PKC-ε were obtained from Santa Cruz Biotechnology (Santa Cruz, CA), anti-GSS, anti-GPX4 and anti-TF were obtained from Proteintech (Rosemont, USA), anti-PKC-α, anti-PKC-δ, anti-p-PKC-ε and anti-pan-PKC were purchased from Bioworld Technology (MN, USA), anti-p-Nrf2 was purchased from Affinity Biosciences (OH, USA), anti-Keap1 was purchased from Wanleibio (Shenyang, China), anti-SLC7A11 was purchased from ABclonal (Wuhan, China), anti-4-HNE was purchased from Bioss (Beijing, China) and anti-NeuN was purchased from Abcam(MA, USA). HRP-coupled second antibodies were purchased from Bioworld Technology (MN, USA). FITC- and Cy3-conjugated secondary antibodies were obtained from Servicebio Biotechnology (Wuhan, China).

Cell nuclear and cytoplasm protein extraction

Cell nuclear and cytoplasm protein extracts were isolated using a Nuclear Protein Extraction kit (Beyotime, China) in according with the manufacturer's instructions. Cells were harvested in cytoplasmic protein extraction buffer A, and added cytoplasmic protein extraction buffer B, centrifugated at 2000 g for 5 min at 4°C. The supernatant was collected as the cytoplasmic fraction. The precipitate was dissolved in nuclear protein extraction reagent on ice for 30 min, centrifugated at 12,000 g for 10 min at 4°C. The supernatant was collected as nucleus fraction.

Nissl staining

For Nissl staining, brain sections were de-paraffinized with xylene followed by rehydration. Sections were stained with 0.5% toluidine blue staining (Servicebio, China) for 5 min, and differentiated with 0.1% glacial acetic acid for 30 s. After rinsed with water and dried, neutral resin was used for sealing the sections. Nissl-positive neurons were quantified in the cortical tissues of lesioned hemisphere by microscopy (Olympus, Japan).

Transmission electron microscopy analysis

Transmission electron microscopy (TEM) was used to visualize the mitochondrial morphology in cerebral cortex. Rats were anesthetized by intraperitoneal injection of 1% pentobarbital sodium, and brain tissues ($1mm^3$) were harvested and fixed in 2.5% glutaraldehyde (Servicebio, China) overnight at 4°C. Tissues were postfixed in 1% OsO4 for 2 h at room temperature, dehydrated in a graded series of acetone, and embedded in epoxy resin at 37°C for 12 h. After heat polymerization of the resin at 60°C for 48 h, the sections were cut (65-70 nm thickness) and then observed under a transmission electron microscope (Hitachi, Japan).

TTC staining

2,3,5-triphenylterazolium chloride (TTC, Sigma Aldrich, USA) was used to evaluate the infarct volume after MCAO/R. Twenty-four hours after reperfusion, brain tissues were removed and placed at -20°C for 30 min, and were sliced into 2 mm intervals. Brain sections were stained with 0.2% TTC (Sigma Aldrich, USA) at 37°C for 5 min. Infarcted areas were analyzed using Image J (Fiji) software. We estimated the infarct volume as a percentage of the ipsilateral the following formula: [(volume of contralateral-volume of non-infarcted ipsilateral)/volume of contralateral] × 100%.

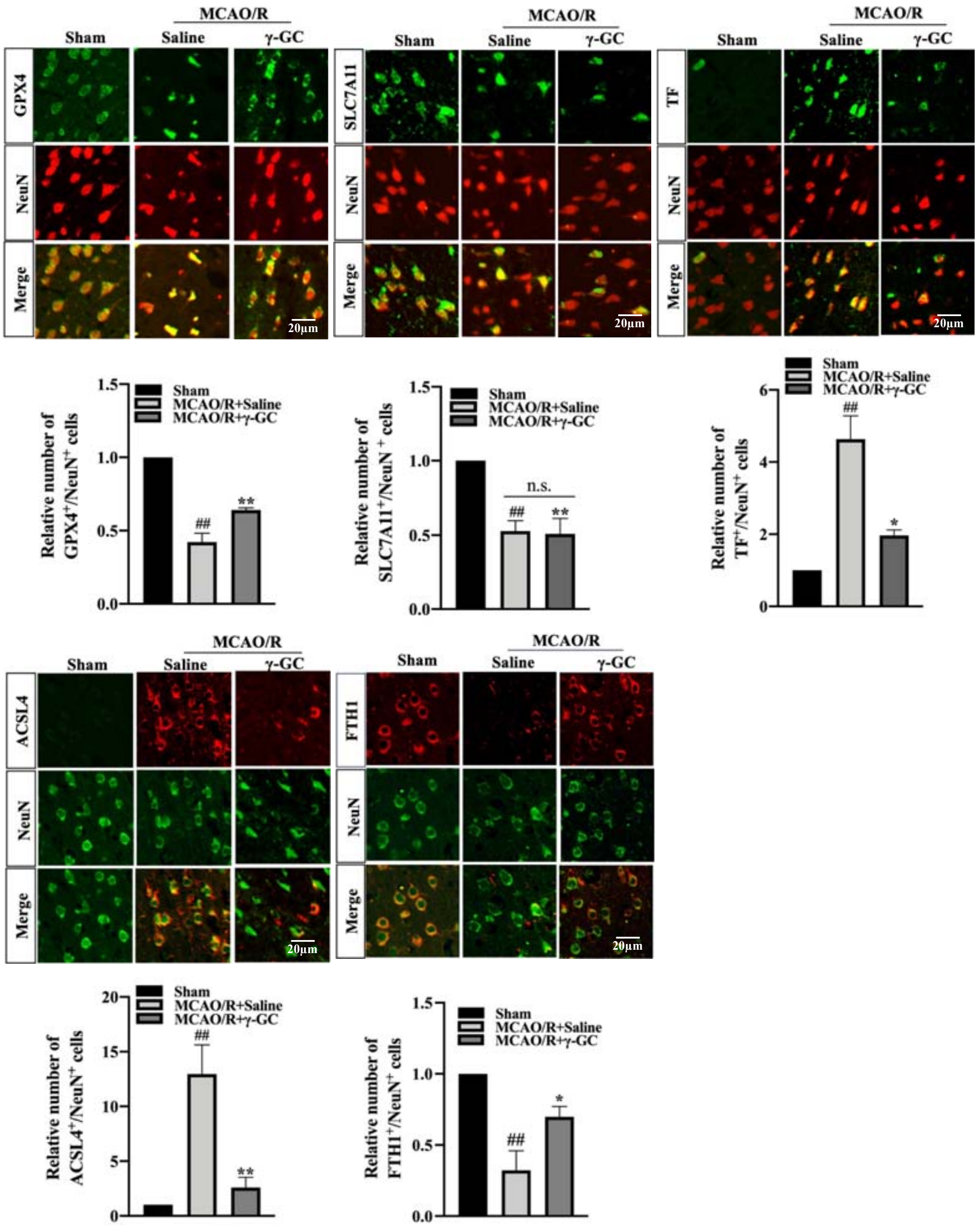

Figure S1. Effects of γ-GC on ferroptosis related protein levels after MCAO/R. Rats were treated with γ-GC (688mg/kg body weight) 1.5 h after being subjected to MCAO. Twenty four hours after reperfusion, the protein levels of indicated protein in cortex tissues were detected by immunofluorescence assays. Data are showed as mean ± SD (n = 3, 3 rats/group), ##p<0.01 *versus* sham group; *p<0.05, **p<0.01 *versus* MCAO/R group.

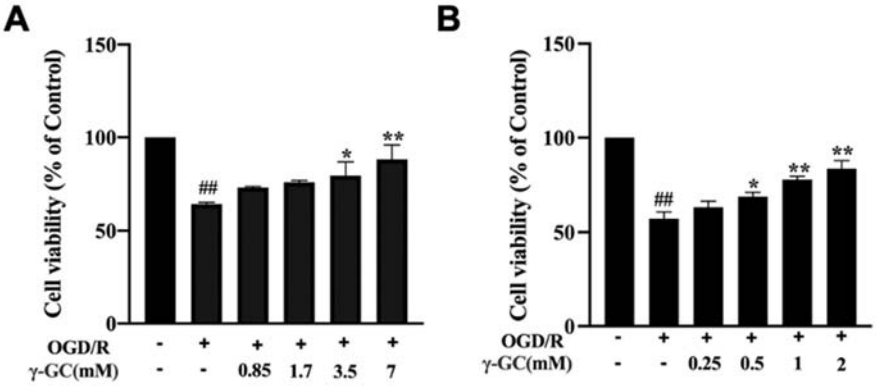

Figure S2. Effects of γ-GC on neuronal cell viability. (A) PC12 cells were treated with indicated concentration of γ-GC after OGD. Twelve hours after reoxygenation, and cell viability was evaluated by CCK-8 assay; (B) Primary cortical neurons were treated with indicated concentration of γ-GC after OGD. Twelve hours after reoxygenation, and cell viability was evaluated by CCK-8 assay. Data are showed as mean ± SD (n = 6), ##p<0.01 *versus* control cells, *p<0.05, **p<0.01 *versus* OGD/R treated cells.

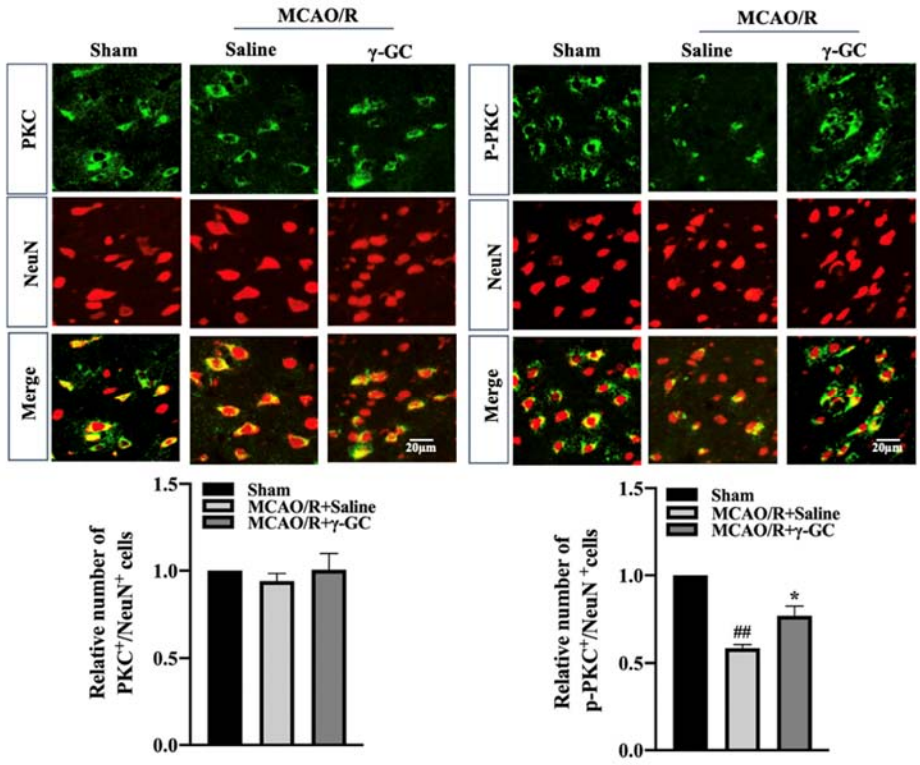

Figure S3. γ-GC increased the level of phosphorylated PKC in cortical neurons after MCAO/R. Rats were treated with γ-GC (688mg/kg body weight) 1.5 h after being subjected to MCAO. Twenty four hours after reperfusion, the protein levels of indicated protein in cortex tissues were detected by immunofluorescence assays. Data are showed as mean ± SD (n = 3, 3 rats/group), ##p<0.01 *versus* sham group; *p<0.05 *versus* MCAO/R group.

Table S1 Primer sequences for qPCR.

Target genes	Accession number	Oligonucleotide primers (5′-3′)	Product length (bp)
Acsl4	NM_053623	Forward 5′-CCTTGGCAAAGAAGCTGTCG-3′<br>Reverse 5′-TCGGGGTACTCCGCTCTATT-3′	171
Gpx4	NM_001039849	Forward 5′-CGATACGCCGAGTGTGGTTT-3′<br>Reverse 5′-GGCATCGTCCCCACTTACAC-3′	156
Fth1	NM_012848	Forward 5′-CAGCGAGGTGGACGAATCT-3′<br>Reverse 5′-TTCACACTCTTTTCCAAGTGCAG-3′	110
Tf	NM_001013110<br>NM_017055	Forward 5′-CCGTGGTGAAGAAGGGAACA-3′<br>Reverse 5′-GACACAACTGCCCGAGAAGA-3′	182
Slc7a11	NM_001107673<br>XM_001053328	Forward 5′-TGCCCGGATCCAGATTTTCC-3′<br>Reverse 5′-CCAAGGGCAACCCCATTAGA-3′	152
Gss	NM_012962	Forward 5′-TTCCGAGATGGCTACATGCC-3′<br>Reverse 5′-CCCCTTCTTCACCCATGTCC -3′	247
β-actin	NM_031144	Forward 5′-CTGAGAGGGAAATCGTGCGT-3′<br>Reverse 5′-CTTCTCCAGGGAGGAAGAGGA-3′	104
